# Periodontal Health and Disease in the Context of Systemic Diseases

**DOI:** 10.1155/2023/9720947

**Published:** 2023-05-13

**Authors:** Gaetano Isola, Simona Santonocito, Saturnino Marco Lupi, Alessandro Polizzi, Rossana Sclafani, Romeo Patini, Enrico Marchetti

**Affiliations:** ^1^Department of General Surgery and Surgical-Medical Specialties, School of Dentistry, University of Catania, Catania, Italy; ^2^Department of Clinical Surgical, Diagnostic and Paediatric Sciences, University of Pavia, Pavia, Italy; ^3^Institute of Dentistry and Maxillofacial Surgery, Fondazione Policlinico Universitario Agostino Gemelli, Catholic University of the Sacred Heart, Rome, Italy; ^4^Department of Life, Health and Environmental Sciences, University of L'Aquila, L'Aquila, Italy

## Abstract

During recent years, considerable progress has been made in understanding the etiopathogenesis of periodontitis in its various forms and their interactions with the host. Furthermore, a number of reports have highlighted the importance of oral health and disease in systemic conditions, especially cardiovascular diseases and diabetes. In this regard, research has attempted to explain the role of periodontitis in promoting alteration in distant sites and organs. Recently, DNA sequencing studies have revealed how oral infections can occur in distant sites such as the colon, reproductive tissues, metabolic diseases, and atheromas. The objective of this review is to describe and update the emerging evidence and knowledge regarding the association between periodontitis and systemic disease and to analyse the evidence that has reported periodontitis as a risk factor for the development of various forms of systemic diseases in order to provide a better understanding of the possible shared etiopathogenetic pathways between periodontitis and the different forms of systemic diseases.

## 1. Introduction

In recent decades, significant progress has been made in the comprehension of the pathogenesis and pathophysiology in periodontal diseases (PD), their interaction with the host, and their relationships with systemic diseases [1].

Periodontal research has identified specific microbial pathogens (consisting of anaerobic Gram-negative bacteria such as *Aggregatibacter Actinomycetemcomitans* (*A. a.*), *Porphyromonas gingivalis* (*P. gingivalis*), *Tannerella forsythia* (*T. forsythia*), *Treponema denticola* (*T. denticola*), and *Spirochetes*) which, in conjunction with mildly virulent organisms, highly organised complex communities in the form of biofilms, is presented first at the supragingival level and, in the more advanced stages, the subgingival [2]. The presence of periodontal pathogenic biofilm induces inflammatory and immune mechanisms of the host with the following inflammatory response and tissue alteration that occurs in the gingival tissues and tooth support apparatus (including alveolar bone) [3, 4]. An important role in the inflammatory response by the host immune system, which can change from one individual to another, is represented by exposure to certain risk factors [4, 5]. Multiple host risk factors associated with the multifactorial etiology of periodontitis are recognised. In recent decades, these risk factors have been studied in-depth and include genetic predisposition to the disease, associated with certain environmental stimuli (diet, diabetes, smoking, poor oral hygiene, etc.) that have been closely associated with the development and progression of periodontitis. In the new classification of periodontal diseases, drawn up in 2017, risk factors assume a relevant role in both diagnosis and prognosis definition for each patient. It has introduced the concept of “risk stratification,” which is based on well-validated risk factors, smoking and uncontrolled type II diabetes. The presence of which in the patient's history increases the probability of the case progressing at a faster rate than is typical for the majority of the population or responding less predictably to standard therapy. Therefore, diabetes and smoking represent “grade modifiers” in that, if present, they are responsible for the progression to a higher degree, independently of the grade resulting from the analysis of direct evidence loss of clinical attachment loss (CAL) in the last 5 years and indirect evidence of progression (age-related bone loss and phenotype) [6].

From there, the inflammatory response during periodontitis has been shown to be marked by the local release of specific proinflammatory mediators and enzymes, including C-reactive protein (CRP); metalloproteinases (MMPs); interleukin- (IL-) 1*β*, IL-6, and IL-10; and tumor necrosis factor (TNF-*α*). It has been widely demonstrated that the periodontal damage associated with periodontitis, with the development of deep periodontal pockets, represents an extremely favourable microenvironment for anaerobiosis with a relative increase in inflammatory cytokine levels associated with periodontal destruction. Given the cumulative increase in inflammatory cytokines, periodontitis usually evolves into acute and chronic inflammatory status at the gingival level, which can represent a negative factor and possible risk factor for developing various systemic diseases, including diabetes, endocrine diseases, metabolic syndromes, and endothelial and cardiovascular diseases. Based on these associations, the concept of “periodontal medicine,” coined by Stephen Offenbacher, has developed in recent years.

In the last twenty years, several epidemiological, experimental, and interventional studies on large-scale populations have shown how periodontitis may negatively impact systemic health, both in healthy patients and in those suffering from pathologies. Specifically, periodontitis has been independently associated with a large number of chronic noncommunicable diseases related to ageing, premature death, and low quality of life. In the study conducted by Romandini et al. in 2020 [7], the results have also indicated that periodontitis and all types of edentulism are associated with an increased risk of mortality from cardiovascular disease, cancer, coronary disease heart disease, and cerebrovascular disease, but not pneumonia.

Diabetes or cardiovascular diseases are still the most studied systemic diseases associated with periodontitis. There have been more and more trials that have attempted to evaluate periodontitis as a risk factor for the development of different diseases including metabolic syndromes, obesity, rheumatoid arthritis, autoimmune diseases, cognitive disorders (e.g., Alzheimer's disease), and even some forms of cancer, all of which are independently associated with or aggravated by the presence of periodontitis. Only more recently, however, a connection was observed between PD, reproductive health, and fertility problems in men and women, as well as negative pregnancy outcomes such as preterm delivery, preeclampsia, miscarriage, and low birth weight of babies [8]. Although it has been reported that periodontitis is linked to about 60 systemic diseases, there are still many aspects that are not known or sufficiently understood, creating therefore discordant thinking within the scientific community.

In light of the above, the aim of this review is to update the knowledge regarding the association between periodontitis and systemic diseases, trying to improve the understanding of possible shared etiopathogenetic pathways between periodontal disease and systemic diseases in relation to the evidence that has emerged in recent years.

For this purpose, a series of searches were carried out using the main scientific databases, such as PubMed, Scopus, and Google Scholar. The keywords of the searches performed were the following: periodontitis, periodontitis and systemic diseases, periodontitis and cardiovascular diseases, periodontitis and diabetes, periodontitis and osteoporosis, periodontitis and obesity, periodontitis and neurodegeneration, periodontitis and infertility, and periodontitis and adverse pregnancy outcomes. The included studies were selected independently by the authors.

## 2. Periodontitis and Cardiovascular Disease

Globally, noncommunicable diseases (NCDs) are increasing in both prevalence and incidence due to the increasing average age of the population, mainly due to unhealthy diets and lifestyles accounting for over 41 million deaths per year in the world or 71% of all global deaths [9]. Surveys carried out over the last decade have shown that 80% of people over the age of 65 in the USA have one or more NCDs and about 7777% have at least two forms of NCD, with a significant burden of disease that negatively affects both the individual and the economy of the health system. In fact, the presence of the comorbidity of at least two NCDs represents an important challenge for a nation's health system, which can cover over 60% of total health costs [10]; in this regard, in recent years, healthcare spending has mainly focused on general health prevention programs.

Among the main NCDs, cardiovascular diseases (CVDs) represent one of the main causes of death in the world, with an estimated average annual death of 17.7 million people; about 31% of these deaths were CVDs, including stroke, myocardial infarction, or valvular diseases. In order to significantly reduce the incidence of CVD diseases and deaths by 2025, the World Health Organization has already introduced a specific worldwide plan of action [11].

CVD is used as an acronym encompassing atherosclerotic disease, mainly coronary, peripheral, and cerebrovascular diseases. In the medical field, there is now evidence that demonstrates a close association between the presences of specific gene polymorphisms that play a key role in the process of atherogenesis and, in general, in the development of CVD [12]. However, a major stimulus in CVD development is several environmental risk factors, including lifestyle factors, primarily smoking, alcohol, dyslipidemia, impaired glucose metabolism, and hypertension. Among other things, there is a marked association between CVD and metabolic diseases with a bilateral relationship, especially in the presence of diets based on processed carbohydrates, salt, and saturated fats which contribute to obesity, type II diabetes mellitus, and CVD, representing the main risk factors for myocardial infarction [13] and stroke [14]. In this regard, one of the cardinal principles of every form of NCD, especially CVD, is represented by identifying individuals at greater or lesser risk of developing the disease through a preventive reduction of risk factors to reduce the disease burden. The risk factors described above are all modifiable through the initiative of improved lifestyles, including physical activity, proper diet, intake of antioxidants and vegetables, and moderate consumption of alcohol [13].

However, CVDs have also been associated with several chronic inflammatory, infectious, or multifactorial diseases, related to a sharp increase in CVD development, which include preterm birth, psoriasis, systemic lupus erythematosus, rheumatoid arthritis, and also periodontitis [15].

More specifically, an increasing body of evidence has supported the close existence of an independent correlation between periodontitis and different NCDs, including diabetes [16, 17], CVD [3, 18], lung diseases [19], and acute and chronic nephropathies [20]. Indeed, periodontitis is independently associated with the same causes and risk factors as CVD. Among the various mechanisms proposed for CVD development, there are also bacteremia and associated systemic inflammatory sequelae, which are associated with an increase in the host response with the release of C-reactive protein, inflammatory mediators, and associated oxidative stress [21, 22]. In patients with multimorbidity, including diabetes and chronic kidney disease, periodontitis is related to reduced survival with a significant increase in CVD and associated pathologies [23]. Therefore, periodontal disease may not be an easily modifiable risk factor for the development of CVD.

**Figure 1:**
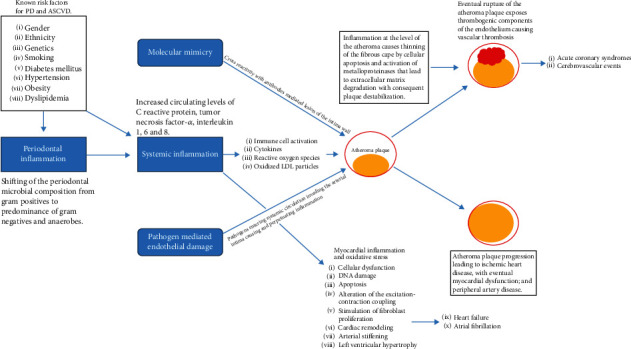
Proposed model for the correlation between CVD and periodontitis. From “Periodontal Disease, Systemic Inflammation and the Risk of Cardiovascular Disease,” Carrizales-Sepulveda [29], Heart, Lung and Circulation 2018, Vol 27 (11): 1327-34, reproduced with permission from Elsevier.

In this regard, a joint workshop was held in 2012 between the American Academy of Periodontology (AAP) and the European Federation of Periodontology (EFP) and a subsequent perio-cardio workshop in 2019 (in order to reevaluate the evidence relating to the association between CVD and periodontal disease) [24]. The workshops highlighted the significant epidemiological evidence that periodontal disease represented a real risk factor for the increased development of atherosclerotic CVD through several mechanisms, including the presence of oral dysbiotic microbiota that can directly or indirectly induce a potential negative systemic inflammation with an impact on the development of atherothrombogenesis [25]. However, even if the inflammation of the periodontal tissue has been correlated in the last twenty years with a higher possible incidence of CVD events, different mechanisms have been hypothesised to explain the correlation between CVD and periodontal disease. Among these are the common status of systemic inflammation that determines these pathologies, the molecular mimicry, and the direct vascular damage associated with the various stages of the disease and mediated by the release of similar inflammatory and microbial agents [1, 26] (Figure 1). Although there are classic risk factors for CVD and periodontitis such as age, sex, hormonal factors, diabetes, hypertension, and hypercholesterolemia, there is nevertheless an important number of acquired factors that can be decisive as a risk factor. In fact, the initial evidence that suggested a correlation between periodontitis and CVD was that hospitalised individuals for acute CVD presented worse dental hygiene more often than healthy patients. Therefore, according to a recent study by Dembowska et al., the prevention and treatment of periodontitis, especially in patients in the so-called high-risk group for cardiovascular disease, are of crucial importance [27].

Since then, further studies and meta-analyses have been carried out, suggesting that the link between CVD and periodontal disease is very high and linked to more complex risk factors and patterns. Among the various studies, Humphrey et al. [28] showed that the presence of moderate to severe periodontitis caused a proportional enhanced risk of coronary heart disease (CHD) approximately three times greater. Specifically, the relative risk (RR) demonstrated that developing CDH in individuals affected by the periodontal disease was between 1.24 and 1.34, while it was also not significantly elevated in patients with gingivitis alone [28]. Other authors have suggested that there is a low correlation between periodontitis and CVD and that an abundant supra- and subgingival biofilm was the primary factor in the development of CVD and associated endothelial damage (Figure 1).

**Figure 2:**
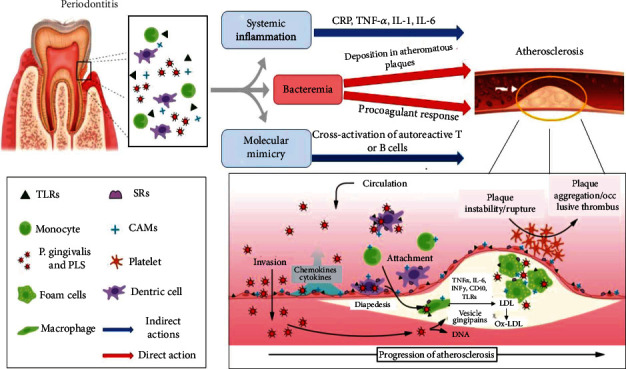
From “Association between Periodontal Disease and Atherosclerotic Cardiovascular Diseases: Relationship between PD and ACVD Induced by Endothelial Dysfunction.”

The studies on the microbiome made it possible to determine better which periodontal pathogens, among the bacteria of the biofilm, were strictly associated with the development of CVD and endothelial damage. Among these, the presence of Gram-negative anaerobic bacteria such as *T. forsythia* and *P. gingivalis* has been related to an enhanced risk of myocardial infarction and endothelial diseases with a 2.52- and 2.99 times higher probability compared to control patients. In fact, the increase in the levels of Gram-negative bacteria during periodontitis determines the capacity to unleash a sustained immune reaction through own pathogenic mechanism, such as lipopolysaccharide (LPS) [2] at the vascular and endothelial level, triggering a subsequent systemic host immune response closest to the deep vascular lumen [30]. Numerous publications showed that periodontal bacteria related to chronic inflammation common among periodontitis and CVD can alter the barrier function of the vascular endothelium by means of a specific epithelial-mesenchymal transition that results in vascular damage [30, 31]. This transition includes cellular events that begin with the loss of cellular structure, adhesion proteins, the extracellular matrix and the epithelial phenotype, and the mesenchymal-like nature of the vascular epithelium which is the basis of endothelial damage and dysfunction. This results in the loss of the epithelial layer's traditional morphological and physiological structure with the formation of vascular microulcerations that facilitate the penetration of periodontal pathogens and associated virulence factors into the vascular circulation [32, 33]. On the other hand, oral biofilm bacteria have also been shown to be present in the formation of atheroma with a high capacity to evade the host's immune response. Various explanations have been provided to explain how periodontal disease adversely affects the development of CVD. The first phase is linked to the direct invasion of the host's endothelial tissues by periodontal pathogens. This theory is supported by polymerase chain reaction studies on atherosclerotic plaques, which demonstrated the presence of Streptococcus mutans (78%), and *A. a.*, *Tannerella forsythia* (*T. forsythia*), *Prevotella intermedia* (*P. intermedia*), and *P. gingivalis* in atheromatous plaques and vascular tissues of patients affected by CVD episodes. However, at present, it has not been established how these bacteria modulate atherosclerosis, possibly due to the capacity of some bacteria, including *P. gingivalis*, to trigger specific T cells or cause a state of secondary inflammation leading to endothelial dysfunction. The most accepted theory of CVD development is that which associates the increase in systemic levels of inflammatory cytokines through an indirect pathway mediated specifically by some periodontal pathogenic bacteria. In fact, a pathogenic biofilm has been associated with the strong release of factors stimulating atherosclerotic vascular diseases, such as cytokines such as IL-1*β*, IL-6, IL-8, and TNF-*α* and the chemotactic proteins of monocytes. Some of these can lead to increased hepatic production and the release of plasma proteins such as fibrinogen and CRP. Furthermore, bacterial components like LPS associated with periodontitis are associated with the strong immune response that could trigger atherosclerosis through their influence on the endothelium with altered lipid metabolism and increased oxidative stress. This was pointed out by the results of various sets of evidence that demonstrated high endothelial dysfunction in individuals with periodontal disease [34, 35] (Figure 2).

Furthermore, the possible criticality of blood sedimentation of bacteria following nonsurgical periodontal therapy was raised. It has been observed that bacteremia frequently occurs immediately after scaling and root planing (SRP). *P. gingivalis* showed the highest frequency of incremented levels in the blood. However, after 30 minutes, bacteria levels in the blood are already decreased. Interestingly, flossing induced higher viridans streptococcal bacteremia compared to SRP, but this difference was not significant. However, it should be noted that this bacteremia condition is transient and the primary cause is oral microbiota. Nonsurgical periodontal therapy is safe in healthy patients. In subjects at risk, prophylaxis with oral antibiotics or irrigation with antiseptic may be indicated to reduce the incidence of bacteremia [36].

However, based on the current evidence, it is difficult to assert with any certainty that periodontitis represents a direct route of inflammation for an enhanced risk of CVD; this in part is due to the fact that patients with periodontitis also have also significant other risk factors such as diabetes and tobacco use, which can be a confounder in the study results [37, 38]. However, data is emerging that periodontitis, even excluding confounders, can significantly determine increased systemic inflammation related to the risk of developing CVD. In this regard, a recent meta-analysis showed increased concentrations of CRP plasma in individuals affected by periodontal disease compared to healthy patients. Also, the treatment of periodontitis induced a significant decrease in CRP levels [39]. Moreover, although several lots of evidence demonstrated a correlation between periodontitis, systemic inflammation, and CVD, some data suggests how periodontitis could be linked to CVD by genetic factors. In this regard, Czesnikiewicz-Guzik et al. [40] evaluated the effect of periodontal disease on blood pressure levels by means of Mendelian randomisation on about 750,000 participants (UK-Biobank/International Consortium of Blood Pressure-Genome-Wide Association Studies), highlighting that some similar single-nucleotide polymorphisms related to periodontitis cause increased blood pressure. In addition, this study in hypertensive patients with moderate to severe periodontitis proved that an active treatment of periodontitis for 2 months resulted in a significant decrease in systolic and diastolic blood pressure of 7.5 and 5.8 mmHg over 24 hours, an inflammatory mediator reduction in association with CVD, and an improvement in flow-mediated dilation, also demonstrating an influence of periodontitis on CVD through specific oxidative stress pathways [40].

### 2.1. Role of Oxidative Stress during PD and Cardiovascular Disease

Oxidative stress is a highly defined mechanism that promotes several inflammatory diseases, including CVD and periodontitis [41–43]. Reactive oxygen species (ROS) cause dysfunction at the cellular and extracellular matrix levels. The oxidation of proteins and lipids can result in specific damage to deoxyribonucleic acid (DNA), causing diffuse cellular apoptosis and necrosis. Furthermore, the various ROS forms cause alteration of the contractile function of muscle cells by modifying specific proteins in muscle contraction, favouring the proliferation of fibroblasts and metalloproteinases with the result of a strong remodeling of cardiac muscle fibers [44]. In this regard, experimental studies on animal models observed a close correlation between periodontitis oxidative stress and cardiac stress since rats that had been induced with periodontitis showed significantly high levels of markers of oxidative heart damage in the left ventricle when compared to rats without periodontitis.

Specifically, the ROS and associated oxidative stress are primarily involved in the onset and progression of various pathological pictures such as periodontitis, Parkinson's disease, and CVD through different modalities [45]. First, as a result of infection from the oral biofilm associated with an important inflammatory reaction on the part of the host, there is the production of ROS proteins. In this regard, evidence has shown that subjects affected by the periodontal disease had significant levels of polymorphonuclear cells that determined a local and systemic increase in ROS when compared to healthy patients [46]. Furthermore, the high oxidative stress status identified in specific phenotypes of patients with periodontitis showed that the high oxidative stress due to the release of ROS at the local (gingival) level due to the periodontopathogenic biofilm could increase the oxidative stress levels and systemic inflammation. Moreover, patients with periodontitis have been shown to have low antioxidant status in crevicular gingival fluid (GCF), with an association between this status and high ROS levels, a key factor in oxidative damage and gingival tissue destruction very similar to tissue damage associated in both patients with periodontitis and CVD. Specifically, the release of ROS induced by infection and oxidative stress due to periodontitis causes greater systemic inflammation, especially at gingival level, making it more susceptible to infections and to the transfer of risk mediators of endothelial damage into the systemic vascular circulation. Furthermore, even a state of chronic inflammation due to altered oxidative stress determines the favouring of highly predictive events of CVD [47], a primary connection between the induction of tissue damage during periodontal disease and systemic inflammation typical of CVD and endothelial damage. At the same time, conditions associated with an unregulated lifestyle together with other comorbidities (e.g., obesity and diabetes) can determine high oxidative stress with an increase in ROS and lipid peroxidation mediators, which may also enhance the individual's susceptibility to the development of periodontitis [48]. Therefore, in patients susceptible to periodontitis, when exposed to LPS and bacterial antigen, infection promotes the recruitment of neutrophils and the synthesis of proteolytic enzymes, which further release ROS from which the gingiva stimulates a negative action of oxidative stress at a local level with consequent tissue damage. However, as moderate to severe periodontitis progresses, the gingival inflammatory status results in ROS production and inflammatory mediators, which thus spread into the systemic bloodstream. For these reasons, there is an increase in oxidative stress on vascular tissue and other districts, causing circulating oxidative stress [49]. This action has been demonstrated by the specific ability of some species of periodontal pathogenic bacteria (e.g., *P. gingivalis*), which suppress ROS detoxification by consuming large quantities of antioxidants present in the gingival tissue, accelerating the inflammatory status and causing oxidative damage and decay of the tooth support tissue [37]. This is related to a progressive change in food intake with a decrease, especially in adolescents, of fruit and vegetable intake related to a concomitant growing intake of soft drinks, which has led to an increase, over time, in the risk of developing periodontitis and CVD [50]. However, associated with altered oxidative stress status, the primary risk factor for CVD in patients with periodontitis appears to be determined by increased CRP levels. CRP plasma concentrations are increased in subjects affected by the periodontal disease due to several studies carried out on populations even on a large scale in different continents; CRP has several biological functions relevant to the pathogenesis of CVD. It is widely demonstrated that CRP is involved in atherogenesis thanks to its capacity to bind to modified low-density lipoproteins promoting endothelial dysfunction, with harmful consequences in the instability of the atheromatous plaque and associated thrombosis. However, although subjects affected by the periodontal disease have increased CRP levels, the effects of periodontal therapy on this marker are variable, especially in patients with concomitant conditions (i.e., diabetes and obesity) which act as strong comorbid factors that contribute to increasing CRP levels [51], even if with not well-defined mechanisms. Conversely, a study conducted on an Indian population showed significant increased CRP plasma concentration in subjects with CVD and periodontal disease than in patients with periodontitis alone, suggesting a synergistic mechanism of increase of this marker by patients with both periodontitis and CVD [52, 53].

Finally, some recent evidence has analysed the thrombotic potential and CVD risk due to platelets and some coagulation mediators. Among these, in addition to platelets, elevated levels of fibrinogen (a highly predictive risk factor for atherosclerosis) and its degradation products are able to stimulate the release of inflammatory mediators and reduce the synthesis of the plasminogen activator inhibitor, which is a major marker of fibrinolysis inhibition, with the risk of thrombus development [54]. Furthermore, recent evidence has proved increased fibrinogen levels in subjects affected by the periodontal disease [55, 56], also noting that the platelet count in patients with periodontitis was elevated compared to that of healthy individuals [57] and that periodontal therapy significantly reduced the platelet count [58].

Therefore, the current evidence established that subjects affected by periodontal disease present a high risk of developing CVD through various pathways; however, the increased risk of developing adverse CVD events occurs in individuals with combined periodontitis and CVD. It is also recommended, on the basis of the current evidence, that patients with periodontitis or with CVD should be included in specific periodontal treatment maintenance programs that could reduce the risk of CVD through the significant reduction of oxidative stress, release mediators associated with cardiovascular risk or factors related to thrombogenesis or atherosclerosis. Recently, Marfil-Álvarez et al. conducted a cross-sectional and analytical study that observed that the extent and severity of periodontitis are positively associated with the extent of AMI (acute myocardial infarction) as measured by serum troponin I and myoglobin levels. However, this is still a preliminary study that needs to be further investigated, which through its preliminary data provides further confirmation of the association between PD and AMI [59].

## 3. Periodontal Health and Disease, Diabetes and Chronic Inflammatory Conditions, and Obesity

Periodontal disease is characterised by periodontal tissue destruction, which is a combined result of a deranged immune response to an organised dysbiotic biofilm, and it has been associated with numerous systemic conditions and diseases [37].

**Figure 3:**
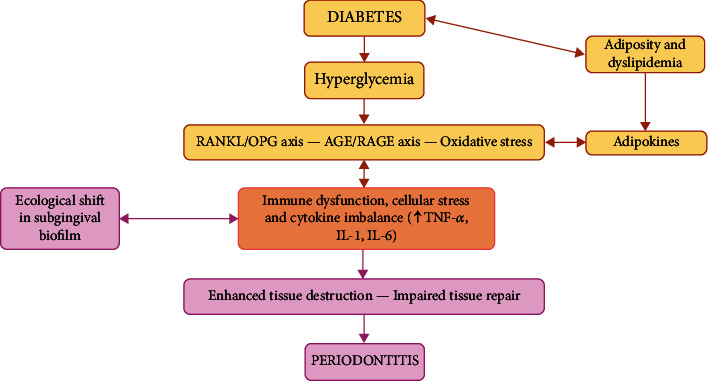
The hyperglycaemic state in diabetic patients induces: (1) the expression of irreversible advanced glycation end products (AGEs) and related receptors (RAGE) and (2) enhancement of oxidative stress and (3) modulation of the RANKL/OPG ratio both directly and indirectly through the AGE/RAGE axis. The result is immune cell dysfunction and cytokine imbalance. All the above, complemented by the effects of subgingival dysbiosis and the circulating adipokines produced due to diabetes-associated adiposity and dyslipidemia, induce a vicious cycle of enhanced periodontal destruction and impaired tissue repair, leading to acceleration and worsening of periodontal disease. Of course, a significant interindividual variation in these processes must be considered (genetics, age, smoking, and stress). Adapted from “A Review of the Evidence for Pathogenic Mechanisms that May Link Periodontitis and Diabetes,” Taylor, J.J. [60], Journal of Clinical Periodontology, 2013, 40: S113-S134, Copyright (2013), reproduced with permission from Wiley.

For many years, the correlation between diabetes and periodontitis has been established. Patients affected by diabetes (both type 1 and type 2) are more susceptible to developing periodontitis, and, vice versa, people with periodontitis show an increased predisposition to diabetes, constituting a “two-way” relationship [60]. In detail, both type 1 diabetes and type 2 diabetes present the same bidirectional relationship with periodontal disease. In fact, whatever the pathogenetic cause of diabetes, autoimmune, or insulin resistance, the result is that there are high glucose concentrations in the bloodstream, with all the consequences that this implies for the correct function of the organism. In 2013, during the European Federation of Periodontology (EFP) workshop, a review explaining the biological plausibility of the bidirectional interrelationship was published [60]. It has been shown that multiple mechanisms may be involved in this sense (Figure 3). Recently, a meta-analysis found that diabetes complications are more frequent in subjects affected by periodontal disease than those without comorbidity. The authors also concluded that patients with periodontal disease have an increased risk of developing type 2 diabetes compared to healthy subjects [61].

**Figure 4:**
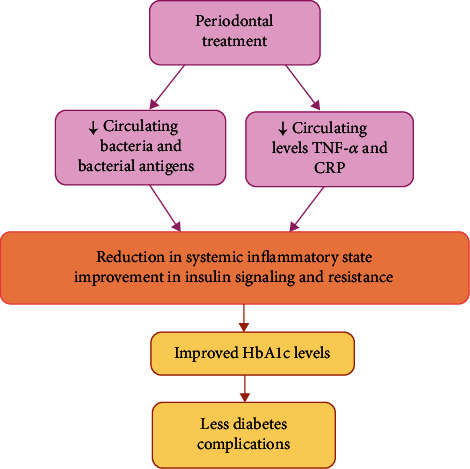
Scaling and root planing determines a reduction of circulating levels of proinflammatory proteins, cytokines, bacteria, and their antigens, thus inducing a lowering of the systemic inflammation and an improvement in insulin signaling and resistance. The reduction in HbA1c levels is a protective factor against the complications of diabetes. Adapted from “An Update on the Evidence for Pathogenic Mechanisms that May Link Periodontitis and Diabetes,” Shapira L. & Polak D. [62], Journal of Clinical Periodontology, 2017, 45: 150-166, Copyright (2017), with permission from Wiley.

Furthermore, several systematic reviews and meta-analyses revealed moderate evidence supporting that periodontal treatment may modestly improve the levels of circulating mediators related to glycemic control in diabetic subjects [55–57, 62–66]. The potential mechanisms are illustrated in Figure 4.

Periodontitis is also influenced by other lifestyle factors such as diet, physical activity, and obesity [4]. Obesity is defined by the World Health Organization (WHO) as an abnormal fat accumulation which constitutes a risk factor for well-being, and it is primarily diagnosed through the body mass index. It is considered a chronic metabolic disease defined by an inflammatory response of the adipocytes associated with the release of hormones and cytokines (adipokines) which can cause changes in blood pressure, dyslipidemia, insulin resistance, and a continuous state of oxidative stress [67]. Obesity has been strongly associated with diabetes, CVD, osteoarthritis, and periodontitis [67, 68].

Many pathogenic mechanisms may link obesity and periodontitis. The promoted proinflammatory state in obese patients may increase the susceptibility to pathogenic bacteria in periodontal tissues. Therefore, obesity can be a modifying factor for periodontitis [68, 69].

Moreover, gingival inflammation may be induced/aggravated by the increased levels of circulating ROS in obese individuals [70]. Two recent systematic reviews supported the negative influence of obesity on periodontitis onset, progression, and response to therapy [71, 72]. Conversely, periodontitis is also associated with proinflammatory cytokines release and, consequently, with other chronic diseases, such as obesity [67]. A recent systematic review found that compromised masticatory function (tooth loss, a principal consequence of periodontal disease) is associated with obesity [72].

**Figure 5:**
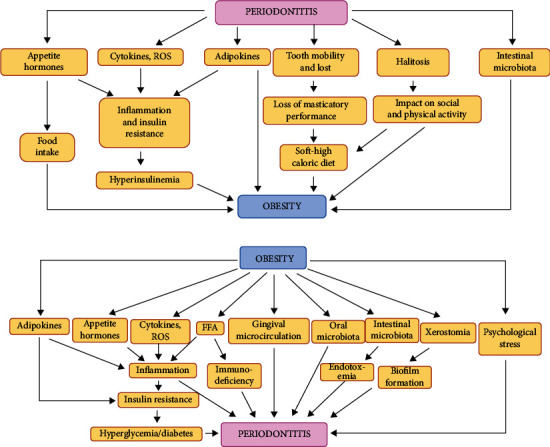
Potential mechanisms linking periodontal disease to obesity (a) and vice versa (b). FFA: free fatty acids; ROS: reactive oxygen species. Modified from “The Association of PD with Metabolic Syndrome and Obesity,” Jepsen S. [74], Periodontology 2000, 2020, 83: 125-153, Copyright (2020), with permission from Wiley.

Furthermore, the results of an experimental study showed that a combination of obesity and periodontitis could have a negative synergistic effect on systematic inflammation resulting in metabolic dysregulation [73]. Therefore, obesity may be a risk factor for periodontitis via the induction of an inflammatory and hyperoxidative state. On the other hand, the destruction of periodontal tissues promotes the release of bacterial antigens and proinflammatory cytokines into the bloodstream, concurring to the development of both inflammatory diseases.  Figure 5 resumes the potential reciprocal mechanisms of the interrelationship between obesity and periodontitis. Finally, interesting studies have shown a decrease in the risk of periodontitis in adults who perform high levels of physical activity. However, further prospective studies are needed to evaluate these aspects better.

Diabetes can be preceded by metabolic syndrome, a condition defined by a spectrum of metabolic abnormalities (obesity, dyslipidemia, dysglycemia, and hypertension) and associated with an enhanced risk for diabetes and CVD [74, 75]. Metabolic syndrome induces a proinflammatory state and has been plausibly correlated to obesity and periodontitis [75]. Three systematic reviews agreed on the possibility of a positive correlation between periodontal disease and metabolic syndrome, but the extent is not clear [76–78]. Further studies are needed to establish causality or to characterise the direction of this association much better.

## 4. Periodontal Health and Disease, Osteopenia, Osteoporosis, and Alveolar Bone Loss

In recent years, many studies have been conducted to understand whether there is a link between periodontal disease and osteoporosis. Both are slowly progressive diseases that share several common features: bone loss. In detail, osteoporosis is a disease characterised by a weakening of the microarchitecture of bone tissue and low bone mineral density (BDM), which induces excessive bone fragility and, consequently, an increased risk of fracture [79]. Currently, the scientific community does not have a unanimous view on the possible link between the two diseases. Choi et al. [80] and Mongkornkarn et al. [81] showed a positive relationship between osteoporosis and periodontitis, while Marjanovic et al. [82] did not observe any clear link. According to several authors, systemic bone loss can significantly influence periodontal destruction. In fact, it is known that oral dysbiosis can induce a rapid resorption of alveolar bone and, consequently, periodontal destruction. However, changes in local tissue responses induced by systemic mediators involved in bone remodeling would also play an important role. RANKL, TNF-*α*, IL1-*β*, and other cytokines, implicated in both the pathogenesis of periodontal disease and osteoporosis, would stimulate the continuous production of osteoclasts by progenitor cells, initiating bone destruction and inflammation. The systemic increases in these cytokines stimulate local osteoclast activity, promote clinical loss of adhesion and destruction of alveolar bone, and accelerate the development of periodontal disease. In addition, poor smoking habits, low calcium intake, vitamin D deficiency, sex, genetics, lifestyle, menopause, and inflammation could increase BMD reduction and the risk of periodontal disease. This has been confirmed by a recent review, which considers that, from the analysis of the current literature, osteoporosis can be listed as one of the risk factors for periodontitis [79]. Osteoporosis and osteopenia are very common conditions in postmenopausal women (with a prevalence of up to 50%), and the postmenopausal state is associated with an increase in severity of periodontitis, with a prevalence of up to 30%. The promotion of alveolar bone resorption and, therefore, the increased severity of periodontal disease in affected women is associated with the role of oestrogen. Oestrogen deficiency promotes both the development of osteoporosis and periodontal disease. More specifically, gum tissue affected by periodontal disease expresses higher levels of RANKL and lower levels of OPG. Furthermore, confocal microscopy showed that 50% of T-lymphocytes and 90% of B-lymphocytes expressed RANKL in diseased gingival tissue and that these percentages were lower in healthy gingival tissue. Oestrogen reduces cytokine production by T cells (TNF*α* and RANKL), monocytes (IL-1 and TNF-*α*), and bone marrow stromal cells (IL-6, RANKL, GM-CSF, and M-CSF); increases TGF-*β* production by osteoblasts; and decreases osteoclast activity and differentiation. In menopause, oestrogen deficiency increases TNF-*α* and RANKL production by T cells, inducing increased osteoclast differentiation; it promotes osteoblast apoptosis. Therefore, periodontal disease and alveolar bone loss are likely to be more severe in menopause, and hormone replacement therapy may prevent or slow the course of periodontal disease and osteoporosis [83].

Furthermore, in a pathological condition, such as periodontal disease, the onset of a disturbance in the homeostasis of bone turnover results in destructive osteolytic processes. These mechanisms are mediated by both bacterial and host-derived factors. More specifically, proinflammatory cytokines such as TNF-*α*, IL-1, and IL-6 have been implicated in the activation of osteoclastic bone resorption in periodontitis. However, many other mediators have been identified that can stimulate osteoclast-mediated bone resorption: IL-11, IL-17, TNF-*β*, TGF-*β*, kinins, and thrombin. Furthermore, studies demonstrated that gingival crevicular fluid contains mediators such as IL-1*α*, IL-1*β*, and PGE_2_ capable of stimulating bone resorption. This fluid is a potential source of extracellular matrix-derived biological markers of alveolar bone resorption in periodontitis [84].

## 5. Periodontal Health and Disease and Cancer

In the field of oncology, an innovative field of research is the possible relationships between dysbiosis, chronic inflammation, and tumors [85, 86]. Many tumors have been related to chronic periodontitis, such as head and neck cancers, colorectal cancer, breast cancer, and pancreatic cancer. Although the biological mechanisms are still not understood, according to the most accepted hypotheses that explain the correlation between periodontal disease and cancer, persistent infection and inflammation related to periodontal disease may cause a critical stimulus to chronic systemic inflammation [87–89].

Head and neck cancers (HNC) may involve different surfaces such as nasal cavities, paranasal sinuses, the pharynx, oral cavity, and salivary glands and are the sixth most malignant tumors [90, 91]. Currently, the connection between the oral biofilm and the up- and downregulation of prooncogenic pathways is poorly understood [85, 86, 92]. However, oral microbiome is involved in establishing and evolving potentially malignant oral and malignant disorders [92]. For example, oral microbiome contributes to ethanol metabolism: the consequent formation of acetaldehyde has toxic effects for epithelial cells, increasing the risk of HNC and especially oral squamous cell carcinoma (OSCC) [86]. Another mechanism might involve Gram-negative bacteria lipopolysaccharides (LPS) that showed higher levels in cancerous conditions [93]. The LPS/TLR4 (Toll-like receptor 4) interaction induces inflammation and type 1 polarisation in T helper cells, suppressing IL-10 expression whose signaling deficiency increases the risk of carcinogenesis [94–96]. Furthermore, oral microbiota composition is different between healthy and OSCC patients. Genera such as *Streptococcus*, *Veillonella*, and *Rothia* are less present in cancerous tissues, while different commensal species including *Fusobacterium nucleatum* (*F. nucleatum*), *P. intermedia*, *Aggregatibacter segnis*, *Peptostreptococcus stomatis*, and *Catonella morbi* are expanded, indicating that they might be opportunistic bacteria with possible relationships with OSCC [93]. Even salivary counts of *S. mitis*, *Prevotella melaninogenica*, and *Capnocytophaga gingivalis* are increased in OSCC patients compared to healthy ones. Recent studies showed that important bacterial species involved in periodontal diseases, such as *F. nucleatum* and *P. gingivalis*, may be correlated to OSCC pathogenesis through different mechanisms: the transformation of normal epithelial cells into cancerous cells through FadA, Fap2, and LPS [97], the promotion of OSCC cell invasiveness via the upregulation of IL-8 and MMPs [98, 99], the induction of epithelial-mesenchymal transition (EMT) of oral keratinocytes by increasing phospho-GSK3ß [100], and the promotion of autophagy processes [100, 101]. Two hypotheses may explain oral microbiota alterations in neoplastic tissues: (1) neoplastic tissues drive alterations in the oral biofilm, or (2) oral microbiota is able to shift towards facilitating carcinogenesis [102, 103]. A meta-analysis that considered five eligible studies evaluated the risk of OSCC in subjects affected by periodontitis, concluding that these individuals showed enhanced susceptibility to oral cancer [104]. A similar systematic review, which selected 12 case-control studies, found that periodontitis is related to a small but significantly higher risk of OSCC; however, this correlation was attenuated after adjusting for smoking and alcohol use [105]. Therefore, it is possible to affirm a plausible correlation between periodontal disease and OSCC; however, the strength of this correlation and the molecular pathways underlying this correlation are not fully elucidated.

With regard to management, recent studies indicate that malignant cells exposed to inflammatory signals develop chemoresistance and more aggressive biological behaviours, promoting tumor progression [106]. Therefore, dentists must look after patients affected by periodontal disease, especially smokers and consumers of alcohol and must also be encouraged to improve domiciliary and professional oral hygiene (scaling and root planing), food hygiene, and in smoking cessation [107].

Breast cancer is the most widespread malignant tumor in women and the sixth cause of cancer-related deaths [108]. The incidence is increasing because of the shorter periods of breastfeeding, later age of first pregnancy, later menopause, an earlier age of menarche, fewer pregnancies, lack of physical activity, alcohol consumption, and obesity [109, 110]. Two longitudinal studies, using data, respectively, from 1676 and 7373 women for a follow-up period of 18 and 6.7 years, reported an enhanced risk of breast cancer in patients affected by periodontitis [111, 112]. A case-control study adjusted for age and smoking status, but with a small sample, found a significant association between periodontal disease and breast cancer. However, other important studies did not find this association [113, 114]. Two recent meta-analyses concluded that periodontitis may increase the risk of breast cancer; therefore, periodontal therapy should be encouraged in the prevention of this tumor [115, 116]. However, additional studies are recommended to confirm these results and reach a consensus.

Some other cancers possibly related to periodontitis are pancreatic cancer and colorectal cancer (CRC). The gut microbiota of colorectal cancer-affected patients show a different composition compared to healthy patients, partially due to ectopic colonisation from bacterial species of the oral microbiota. Evidence for *F. nucleatum* (Fn) involvement in CRC stands out [117, 118]. In particular, this bacterial species is able to favour the growth, migration, and invasion of colorectal cancerous cells, thereby increasing IL-8/chemokine secretion [119, 120] and their resistance to chemotherapy by modulating autophagy [121]. Furthermore, a 10-year follow-up study based on 68273 adults evaluated the role of periodontal disease as a risk factor for cancer mortality [122]. However, from the study conducted by Mesa et al. [123], there are still unknown aspects that need to be clarified regarding the association between PD and CRC. Certainly, understanding how it colonises the gastrointestinal tract remains a hotly debated topic. There are currently two theories: a direct route through the oro-gastrointestinal tract or an indirect route through the blood. Certainly, what is known is that, in the intestine, bacteria would find an ideal microenvironment to disrupt the balance between the local microbiota and the immune system, resulting in dysbiosis. A dysbiotic gut biofilm can cause intestinal diseases such as inflammatory bowel disease, irritable bowel syndrome, and CRC. Finally, the perpetuation of a proinflammatory environment would lead to a chronic exposure to mediators of inflammation, activation of oncogenes, and development of CRC. The authors found an increased pancreatic cancer mortality in patients affected by periodontal diseases.

Cancer currently represents one of the most challenging worldwide health problems in the contemporary age. Primary prevention, and therefore the knowledge of possible etiological factors, is of fundamental importance prognostically. Therefore, delving into the biological mechanisms and the real impact on cancer of an extremely widespread disease such as periodontitis will be a major challenge for research.

## 6. Periodontal Health and Disease, Infertility, and Adverse Pregnancy Outcomes

In recent years, research has observed how periodontal disease and the bacteremia it causes can have a negative impact on reproduction health and fertility issues in men and women and on adverse pregnancy outcomes, such as preterm birth, preeclampsia, miscarriage, and low birth weight in children [124, 125]. Although little information shows a direct relationship between bad periodontal health and fertility problems, it is well known that systemic bacteremia, caused by subclinical infections, can hamper reproductive function in both sexes [126–128]. Gingival tissues contain receptors for the sex hormones, oestrogen and progesterone, which are susceptible to hormonal imbalances that occur in women during the menstrual cycle, pregnancy, menopause, hormone, and contraceptive therapies. The interplay between receptors and ligands and high metabolic activities induces important modifications in the permeability and underlying microcirculation of gingival capillary vessels.

**Figure 6:**
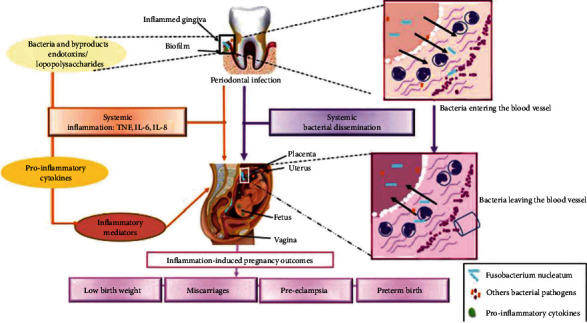
Periodontal disease and adverse pregnancy outcomes. From “Oral Microbiome and Pregnancy: A Bidirectional Relationship,” Saadaoui [135], Journal of Reproductive Immunology, 2021, 2021: 103293, Copyright (2021), with permission from Elsevier.

Such changes may result in an increased inflammatory reaction, suppression of cell-mediated immunity, and changes in the periodontal milieu's microflora, a mechanism which can worsen existing periodontitis [129, 130]. During pregnancy, if there is preexisting gingival inflammation, the metabolism of these hormones in the gum is higher than in normal periodontal tissues [131]. It has been observed that maternal bacteremia, induced by periodontal infection outbreaks, can hinder fetal development and the achievement of pregnancy, as endotoxins and bacterial intermediates in the bloodstream result in bacteremia in the uterus [132]. Therefore, oral bacteria can penetrate and colonise the maternal-fetal unit, thus leading to infertility and gestational disorders [133]. Moreover, a study conducted by Lafaurie et al. recently showed that periodontal disease is an independent risk factor from other important risk factors for an adverse pregnancy outcome (preterm delivery, low birth weight, and preeclampsia), indicating that periodontal disease prevention should be included in preconception and antenatal care programs [134]. Two different mechanisms have been suggested to describe the link between periodontal disease and adverse pregnancy outcomes. The first proposed mechanism is that periodontal bacteria, generated in the gingival biofilm, can move from the oral cavity and enter the intra-amniotic fluid and fetal circulation, through the bloodstream and placenta, directly influencing the fetoplacental unit and thus causing bacteremia. The second mechanism involves the systemic spread of endotoxins and/or inflammatory mediators originating from periodontal bacteria and released from the subgingival inflammatory site, which are transported to the fetoplacental unit via the bloodstream, stimulating an inflammatory response, capable of affecting fetal growth or leading to pregnancy complications (Figure 6) [135].

In a study carried out in Australia by Hart et al., it was observed that women with periodontitis needed about two months more time to reach the required gestation (7.1 months) than women without periodontitis (5 months) [126]. Preterm birth in women with periodontitis is thought to be induced by the systemic spread of oral bacteria and elevated levels of proinflammatory cytokines produced, which are delivered to the systemic circulation in inflammation and cause myocyte contraction and preterm pregnancy. To confirm this, *P. gingivalis* and *F. nucleatum* have been discovered in amniotic fluid samples or in the placenta of mothers with premature birth and periodontitis, while *P. gingivalis* and *A. a.* have been detected in the amniotic fluid of pregnant women with periodontitis [136]. Studies on mice have demonstrated that *P. gingivalis* can have a negative impact on pregnancy: LPS from *P. gingivalis* caused placental and fetal growth restriction and placental resorption [137]. Another study observed that antibodies against *P. gingivalis* caused fetal loss when administered passively [138]. Furthermore, inflammatory mediators produced by periodontal pathogens, released from the circulation or produced by infected endometrial and placental tissues, play a crucial role in the pathogenesis of preterm low birth weight [133]. In several high-quality randomised controlled trials, it has been observed that nonsurgical periodontal therapy carried out during the second trimester of gestation does not improve pregnancy outcomes, because therapy carried out from the fourth to the sixth month of pregnancy is late in preventing placental colonisation by periodontal pathogens and is not able to act on pathogen-induced lesions to the fetoplacental unit sufficiently. This would suggest that intervention during the preconception period could induce more significant improvements [139]. In contrast, a randomised controlled trial found that periodontal treatment was not linked to an elevated risk of preterm labour and could significantly improve periodontal status and lower the risk of preterm birth [140]. Several studies have observed an important connection between periodontitis and the risk of developing preeclampsia, a multisystem gestational disorder characterised by proteinuria and maternal hypertension after 20 weeks of gestation. The connection between the two diseases would again be attributable to the placental inflammatory response resulting from the bacteremia induced by periodontal pathogens migrating from periodontal tissues through the bloodstream. Thus, during infection, oral microorganisms release cytokines and immunoglobulins that limit the growth of pathogens and, at the same time, increase the inflammatory reaction, probably leading to embryonic or fetal lesions [141].

While the association between periodontitis and preterm birth with low birth weight and female infertility is well researched and established, the connection between male infertility and periodontitis is not well understood. Currently, there are few and often conflicting studies available. A possible link between male infertility and periodontal disease has only recently drawn attention. Male infertility can be associated with multiple factors, such as sexual dysfunction or altered sperm quantity and quality. Infections can affect sperm quality and quantity, so periodontitis can affect male fertility. In a 2011 study by Klinger et al. [142], it was observed that profound periodontal pockets and loss of clinical attachment are linked to the submotility of sperm. This finding is supported by the observation of Zhu et al. [143] showing that sperm quality deteriorated with worsening chronic periodontitis. Práger et al. [144] observed that the percentage of participants with dental tartar and bleeding on probing (BOP) is significantly higher among men with idiopathic infertility. A recent case-control study showed a significantly higher prevalence of moderate/severe periodontitis in men with semen abnormalities (case group) compared to the control group of men with normospermia [145]. In contrast to the above-mentioned studies, a study by Pásztor et al. showed that adverse periodontal conditions are not linked to any abnormalities in seminal parameters [146]. The relationship between erectile dysfunction (ED) and PD is endothelial dysfunction. Recently, a study evaluated the prevalence of periodontitis in patients with ED, observing that patients with ED showed a greater extent of moderate or severe chronic periodontitis than the control group [147]. A prospective study, also conducted by the same research group, observed that patients with periodontitis and ED showed a 3.7-fold increased risk of suffering major cardiovascular adverse events after a mean follow-up of 4.2 years [148]. In conclusion, the literature review suggests that preventive actions against periodontal disease in both men and women are justified in order to avoid problems such as infertility and preterm birth.

## 7. Periodontal Health and Disease, Neurological Diseases, and Alzheimer's Disease

Alzheimer's disease is a gradual neurodegenerative disease that results in a progressive and irreversible decline in memory, cognition, language, and learning ability. Cognitive impairment has been associated with the generation of synaptotoxic *β*-amyloid plaques and hyperphosphorylated tau proteins in brain areas linked to enhanced cognitive functions [149]. *β*-Amyloid plaques and neurofibrillary tangles (NFTs) are not pathognomonic of Alzheimer's disease but can be found in other pathological conditions typical of the central nervous system (CNS), including chronic infections, which have these specific histopathological hallmarks. Recent studies also show that *β*-amyloid has significant antimicrobial activity, prompting the idea that infections can prompt its generation and deposition as plaques in the brain [150, 151]. Until a few years ago, the brain was thought to have “immunological privilege” status, but it has been discovered that it can undergo various inflammatory processes, such as the activation of complement, glial cells (microglia and astrocytes) and lymphocytes and the expression of cytokines, chemokines, and reactive oxygen species (ROS), which lead to neuronal apoptosis and dysfunction of the blood-brain barrier (BBB), essential for the integrity and proper working of the CNS, promoting the development of Alzheimer's disease [152]. Thus, neurodegeneration is due both to direct damage caused by *β*-amyloid plaques and tau aggregates and to the innate immune reaction activated to remove these clusters from the brain, which negatively affects neurodegeneration. Inflammation is the connection between periodontitis and Alzheimer's disease, so the inflammatory process associated with periodontal disease could influence the pathogenesis and prognosis of Alzheimer's disease [153]. This has been shown by an elevation of proinflammatory cytokines in older patients with Alzheimer's disease and periodontitis [154]. In a study on mice, it was noted that chronic systemic infection induced *P. gingivalis* led to *β*-amyloid accumulation in the brain, in middle-aged mice, and it also prompted *β*-amyloid accumulation in inflammatory monocytes/macrophages through the activation of CatB/NF-*κ*B signaling [155]. This result is in accordance with findings from a further study in which it was observed that oral infection induced by *P. gingivalis* leads to neurodegeneration and extracellular deposition of *β*-amyloid 42 in the brains of young adult wild-type (WT) mice demonstrating how chronic low-grade periodontal pathogenic infection resulted in the appearance and development of AD-like neuropathology [156]. In 2019, several studies demonstrated a correlation between periodontitis and early cognitive impairment and Alzheimer's disease [149, 157]. In the same year, another study was carried out by Gaur and Agnihotri who observed that *A. a.* activates the secretion of proinflammatory cytokines from microglia [149]. In a cohort study of 219 individuals (110 AD patients and 109 healthy volunteers), conducted by Noble et al. [158], it was found that patients with elevated serum IgG against *Actinomyces naeslundii* (which is linked to periodontal disease) were at increased risk of developing Alzheimer's disease. The authors concluded that periodontitis bacteria are connected to Alzheimer's disease via microbial toxins, inflammatory agents, and serum antibodies. Thus, chronic inflammation developed by these bacteria is a susceptible predictor for the onset of Alzheimer's disease. Lipopolysaccharides of *P. gingivalis* and *T. denticola* have been isolated from the human brain of Alzheimer's disease sufferers, further supporting the hypothesis that virulent elements of these pathogens could have a role in the development of brain inflammation and Alzheimer's disease. Ueda et al. [159] suggest that leptomeningeal cells, which are implicated in the transmission of systemic inflammatory signs between brain-resident macrophages and microglia, secrete inflammatory mediators during periodontitis. In this regard, Kamer et al. [153] observed that Alzheimer's disease patients with periodontitis have an increased level of specific antibodies against periodontal bacteria and TNF-*α*. Similar results have also been reported from further studies with a higher level of TNF-*α* in the serum of patients with AD and periodontal disease [160]. Although the literature review indicates that there is an association between periodontal pathogens and Alzheimer's disease, more longitudinal studies need to be conducted to confirm with certainty that periodontal pathogens and antibodies directed against them are directly involved in neurodegeneration in Alzheimer's disease. However, it should be underlined that bad oral hygiene plays a role in the development of chronic periodontitis and may indirectly enhance the risk of Alzheimer's disease. Conversely, patients with Alzheimer's disease have a limited ability to maintain even little, if any proper oral hygiene or even to see a dentist for the treatment of oral hygiene, which increases the risk of periodontitis but also the progression of Alzheimer's disease [155, 161]. In conclusion, evidence suggests that preserving good oral health can have a preventive effect against Alzheimer's disease.

## 8. Periodontal Health and Disease and Respiratory Disease

During the last decade, the association between periodontal disease and respiratory disorders has been much discussed, and researchers have not yet come to a unanimous opinion. Although several studies claim that proper oral hygiene prevents the development of respiratory diseases, more evidence is needed to verify this [19, 162, 163]. Respiratory diseases and periodontitis are the most common human diseases in the world [164]. Pneumonia, an infectious disease of the lung parenchyma, asthma, and chronic obstructive pulmonary disease (COPD), a chronic condition that includes both bronchitis and emphysema and characterised by airflow blockage caused by an intensified chronic inflammatory response within the airways, may share several immunological processes and etiopathological aspects with periodontal disease [165, 166]. It has been observed that the course of lung disease may be influenced by infective and inflammatory processes like periodontitis [167]. This relationship would be determined by microorganisms in periodontal pockets, especially anaerobic bacteria, which can be drawn into the lower airways, which act as a further inflammatory load in lung tissues. COPD and periodontal disease have comparable pathophysiology, which includes inflammation, recruitment of neutrophils, and release of proteolytic enzymes, leading to pulmonary alveolar disruption or progression of periodontal disease. More studies are needed to confirm a direct connection between COPD and periodontal disease, although several studies report a significant relationship between the two diseases. One study showed that lack of dental care may also be associated with an increased risk of COPD [168]. There have been various systematic reviews evaluating the association between poor oral health and pulmonary diseases—including the one conducted by Ferreira et al. [169] which evaluated the association between periodontal clinical parameters and asthma, the one conducted by Shi et al. [116] who analysing the relationship between worse periodontal conditions and COPD; and the one conducted by Cagnani et al. [162] regarding the association between periodontal disease and pneumonia; these authors concluded that there is an association between periodontal disease and respiratory diseases. These results only consider the association between periodontal clinical parameters, like probing depth (PD), clinical attachment level (CAL), bleeding on probing (BOP) or plaque index (PI), and respiratory disease. In contrast, a recent systematic review found that there is a weak correlation between periodontitis and pulmonary disease [170]. A recent study by Dembowska et al. indicated how inhaled antiasthmatic drugs affect both general and oral health. ICSs (inhaled corticosteroid therapy) worsened BOP at the anterior sextant in patients with asthma but no periodontal disease [171]. In conclusion, further investigation will be required to validate a true association between periodontal diseases and to be able to state with certainty that preventive oral hygiene practices and periodontal care may have a potential future role in decreasing exacerbations of respiratory disease and increasing patients' quality of life.

## 9. Conclusions and Future Directions

Periodontitis is a multifactorial etiology whose pathogenesis depends on the complex interactions between the individual's immune reaction and periodontal pathogens that can evolve in association with specific environmental factors. Although it is agreed that the primary etiology of periodontal disease is infectious in nature due to the supra- and subgingival biofilm, the comprehension of the etiopathogenesis and progression of periodontitis in its various forms has evolved over the years. It has also been well established that factors such as genetics and the role of the immune system contribute significantly to the resistance and susceptibility of the individual to periodontal disease as well as to its rapid or slow evolution.

Recently, research and clinical studies in the periodontal field have made steps forward, with the demonstration of the concept of “periodontal medicine” and which have strongly demonstrated a strict association between periodontitis and oral and systemic health. It is clear that the outcomes of the impact of periodontitis are increasingly considered relevant by health stakeholders and beyond. In this regard, the analysis of oral health-related quality of life measures employed in recent years has highlighted important adequate psychometric properties in terms of reliability and validity to record a change in oral health-related quality in patients with periodontitis and how periodontal treatment is useful for improving both patients' oral and systemic well-being.

Definitive evidence for the role of periodontitis in most chronic systemic diseases is lacking since there have been few well-conducted clinical trials. Moreover, from a careful and in-depth analysis of the various studies in literature, it is not possible to define a strict bilateral association between systemic and periodontal diseases well. However, the research carried out in recent decades in this regard has clearly defined that the comprehension of the etiopathogenesis of periodontitis is translated from the consideration of an exclusively bacterial origin to a more holistic consideration, with a true cause-effect relationship of a multifactorial nature in which genetic, exogenous, and lifestyle factors in general can significantly affect oral health. However, with the ever-increasing understanding of periodontal disease becoming known, our ability to provide “personalised periodontal treatment” is now a fundamental term to address. In the coming years, in order to obtain an effective treatment of periodontal disease, especially in the long term, it will increasingly require a careful analysis of the individual modifiable risk factors such as smoking, diabetes control, and diet, which, through a personalised approach, will surely improve in time the long-term outcomes of the therapies and the quality of patient care at a local and systemic level at the same time. However, large-scale studies in different types of populations will be required to understand the correlation between systemic and PD better and also the impact that periodontitis plays in the etiology, progression, and therapeutic success of systemic diseases.

## Data Availability

Data of the present manuscript is available from the corresponding author upon reasonable request.
